# High Methanol Gas-Sensing Performance of Sm_2_O_3_/ZnO/SmFeO_3_ Microspheres Synthesized Via a Hydrothermal Method

**DOI:** 10.1186/s11671-019-2890-5

**Published:** 2019-02-14

**Authors:** Kun Li, Yinzhen Wu, Mingpeng Chen, Qian Rong, Zhongqi Zhu, Qingju Liu, Jin Zhang

**Affiliations:** School of Materials Science and Engineering, Yunnan Key Laboratory for Micro/Nano Materials and Technology, Yunnan University, Kunming, China

**Keywords:** Methanol gas, Adsorbed oxygen, Specific surface area, p-n heterojunction

## Abstract

In this work, we synthesized Sm_2_O_3_/ZnO/SmFeO_3_ microspheres by a hydrothermal method combined with microwave assistance to serve as a methanol gas sensor. We investigated the effect on the microstructure at different hydrothermal times (12 h, 18 h, 24 h, and 30 h), and the BET and XPS results revealed that the specific surface area and adsorbed oxygen species were consistent with a microstructure that significantly influences the sensing performance. The gas properties of the Sm_2_O_3_-doped ZnO/SmFeO_3_ microspheres were also investigated. With a hydrothermal time of 24 h, the gas sensor exhibited excellent sensing performance for methanol gas. For 5 ppm of methanol gas at 195 °C, the response reached 119.8 with excellent repeatability and long-term stability in a 30-day test in a relatively high humidity atmosphere (55–75% RH). Even at 1 ppm of methanol gas, the response was also higher than 20. Thus, the Sm_2_O_3_-doped ZnO/SmFeO_3_ microspheres can be considered as prospective materials for methanol gas sensors.

## Introduction

Methanol is an important substance in the industry and daily life. It is also an important raw material of many products such as formaldehyde, colors, and antifreeze. Direct methanol fuel cells (DMFC) are considered important alternative fuels for automotive manufacturers that are friendly to the environment [1]. However, methanol can result in total blindness with a dietary intake of 10 mL, and when the amount of methanol is higher than 30 mL, this may cause fatal diseases [2]. Thus, it is necessary to quickly detect low concentrations of methanol gas at lower operating temperatures. However, previous research on methanol gas sensors [3, 4] have not been satisfactory because of the high detection limit (> 50 ppm) and high operating temperature (> 275 °C). In addition, few studies reported on the humidity stability issue of gas sensors.

Metal oxide semiconductors (MOS) play an important role in gas sensors because of their excellent electrical properties. To enhance the gas-sensing performance, some researchers have synthesized semiconductor metal oxides modified with noble metals [5, 6]. However, the high cost and scarcity of noble metals considerably hampers their practical application on a large scale [7]. In recent years, many researchers have focused on constructing heterojunctions, which include p-p [8], n-n [9, 10] and p-n heterojunctions. Due to the chemically distinct components, heterostructures exhibit superior sensing properties compared with single oxides. In particular, the p-n heterojunction is the most common. Li. et al. [11] synthesized a SnO_2_-SnO p-n heterojunction as a NO_2_ gas sensor. The response to 50 ppm NO_2_ gas at 50 °C by SnO_2_-SnO was eight times higher than that of pure SnO_2_. Ju et al. [12] prepared NiO/SnO_2_ as a triethylamine gas sensor, and the response was 48.6, whereas it was 14.5 for pure SnO_2_ at 10 ppm at 220 °C. Qu et al. [7] synthesized a ZnO/ZnCo_2_O_4_ hollow core-shell as a xylene gas sensor. The response of ZnO/ZnCo_2_O_4_ to 100 ppm of xylene gas was 34.26, whereas the response was lower than 5 for pure ZnO.

ZnO is a typical n-type semiconducting metal oxide that has been reported in many research studies in the field of gas sensors because of its convenient synthesis method, low cost, and controllable size [13]. In particular, ZnO has excellent selectivity to alcohol compounds [14–16]. In recent years, researchers have focused on p-type (for example, LaFeO_3_) semiconducting metal oxides in gas-sensing materials because of the high response and good stability [17–19]. In previous studies, SmFeO_3_, which is a typical p-type semiconductor metal oxide, exhibited good sensing, but the sensitivity and stability are still unsatisfactory [20, 21].

**Fig. 1:**
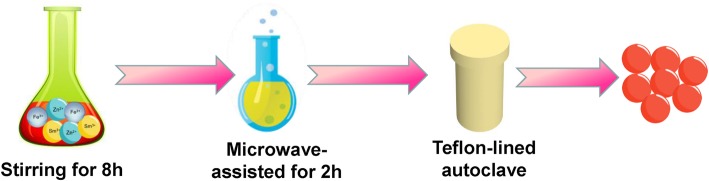
Diagram of the preparation progress of microspheres

In this work, Sm_2_O_3_/ZnO/SmFeO_3_ microspheres were prepared by a hydrothermal method as a methanol gas sensor, and the effect of different hydrothermal times was studied (Fig 1). The gas-sensing results of the Sm_2_O_3_/ZnO/SmFeO_3_ microspheres indicated excellent sensing performance for methanol gas at a relatively low concentration (5 ppm), at a low operating temperature (195 °C), short response (46 s) and recovery (24 s) time, and at a high relative humidity (75% RH) with a high response (119.8). The sensor also displays good repeatability and long-term stability. This excellent sensing performance indicates that Sm_2_O_3_/ZnO/SmFeO_3_ is a promising candidate for sensing methanol gas materials in the future.

## Method Section

### Materials

All the chemicals used in this study were analytical pure grade.

### Synthesis of Sm_2_O_3_/ZnO/SmFeO_3_ Microspheres

The composite was synthesized through a microwave-assisted hydrothermal reaction. First, 4.44 g of samarium nitrate hexahydrate (Sm(NO_3_)_3_·6H_2_O), 4.04 g of iron nitrate nonahydrate (Fe(NO_3_)_3_·9H_2_O), 0.09 g of zinc nitrate (Zn(NO_3_)_2_·6H_2_O), and 4.80 g of citrate were dissolved in 100 ml of distilled water and stirred until the solution became clear. Then, 2 g of polyethylene glycol (PEG) was added. Identical solutions were prepared in quadruplicate. The mixed solution was kept under vigorous stirring at 80 °C for 8 h, and the suspension was placed in a microwave chemical device (CEM, USA) at 75 °C for 2 h. Then, the solution was moved into a Teflon-lined autoclave and heated from 25 °C to 180 °C and maintained for 12 h, 18 h, 24 h, and 30 h at 180 °C. To remove the organics, the obtained iron red precipitate was washed with deionized water several times via centrifugation, and then, it was dried at 60 °C for 72 h and calcined at 700 °C for 2 h. The products, S1 (12 h), S2 (18 h), S3 (24 h), and S4 (30 h), were finally prepared.

### Characterization

The structures of the samples were characterized by using XRD (D/max-2300, Cu Kα1, *λ* = 1.54056 Å, 35 kV). The samples were scanned from 10 to 90° (2θ). The morphology and particle size were examined by field-emission scanning electron microscopy (FESEM). The microstructures of the samples were examined by transmission electron microscopy (TEM) and high-resolution transmission electron microscopy (HRTEM) via a JEM-2100 microscope operating at 200 kV. Energy dispersive X-ray spectroscopy (EDS) was obtained using the TEM attachment. X-ray photoelectron spectroscopy (XPS) was measured on an XPS from Thermo Fisher Scientific Co. Ltd. at 1486.6 eV. The specific surface areas were calculated by the Brunauer-Emmett-Teller (BET) equation based on the nitrogen adsorption-desorption isotherm recorded with a Quadrasorb evo instrument (Quantachrome Co. Ltd.) at 77 K (surface area and porosity system).

### Fabrication and Measurement of Gas Sensors

Gas sensors were fabricated according to the literature [22]. Generally, as-synthesized samples were thoroughly dispersed in deionized water to form a homogeneous paste and then coated onto the surface of a ceramic tube. A Ni–Cr alloy coil heater was inserted into the ceramic tube as a heater to control the operating temperature by adjusting the heater voltage. The gas sensors were aged at 150 °C for 1 week in the air to improve the stability and repeatability of the sensors. The gas-sensing performance of the sensors was measured by a WS-30A gas sensor measurement system. Measurements of the gas-sensing performance were performed in a static system under laboratory conditions.

Gas sensor parameters included the response, selectivity, response and recovery time, and the optimal working temperature. The gas response of a p-type gas sensor is described as:

1 $$ S={R}_{\mathrm{g}}/{R}_{\mathrm{a}} $$

where *R*_g_ represents the resistance in target gases and *R*_a_ represents that in air. Other gases were also tested under the same condition to investigate the selectivity of the gas sensor. The response and recovery time were defined as the time taken by the sensor to achieve 90% of the total resistance change in the case of adsorption and desorption, respectively. Gas adsorption/desorption processes on the surface are largely affected by the working temperature, and the highest response is exhibited at the optimal working temperature.

The concentration of gases obtained by the static liquid gas distribution method is determined by calculating the following:

2 $$ C=\frac{22.4\times \phi \times \rho \times {V}_1}{M\times {V}_2}\times 1000 $$

## Results

### Structural and Morphological Characteristics

**Fig. 2:**
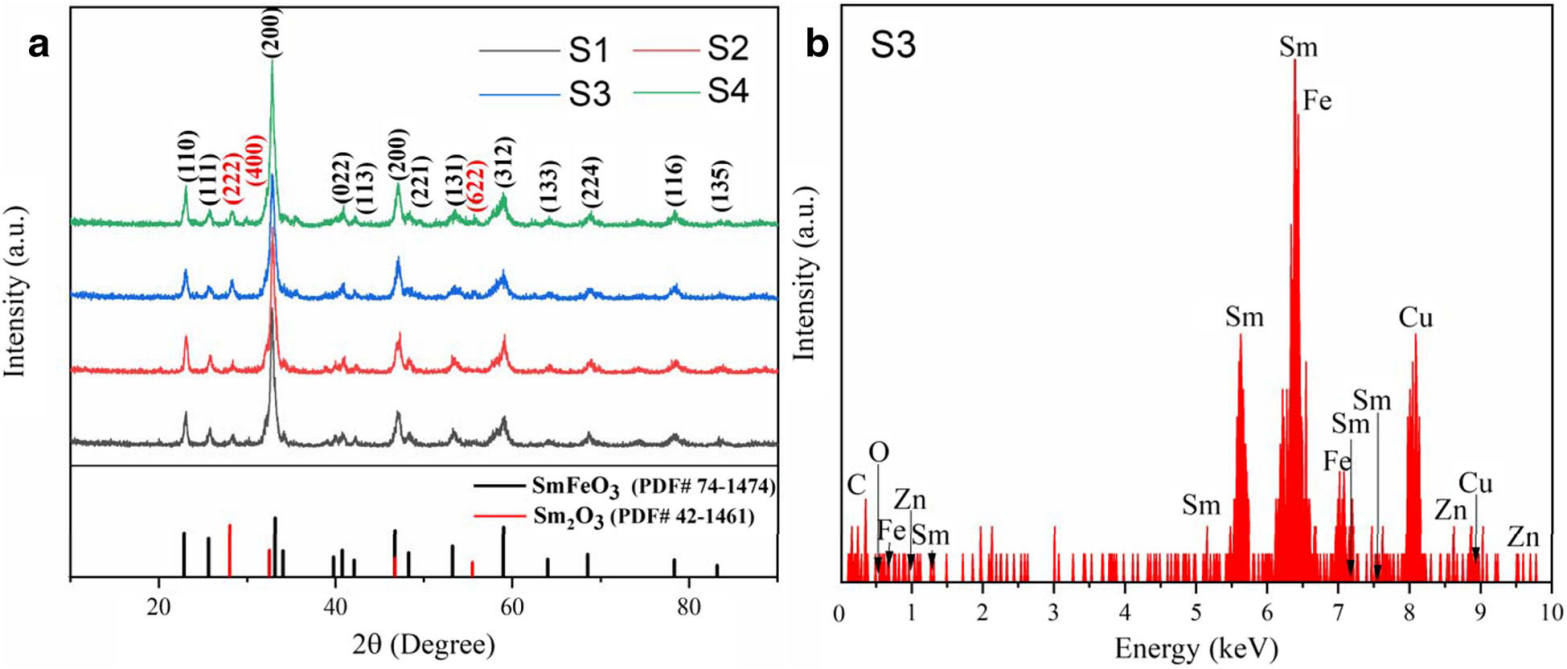
**a** XRD pattern of S1, S2, S3, and S4. **b** EDS spectrum of S3

The X-ray diffraction pattern of as-synthesized S1, S2, S3, and S4 are displayed in Fig. 2a and the corresponding EDS elemental mapping of S3 is shown in Fig. 2b. The main diffraction peaks of the samples obtained with different hydrothermal times are assigned to SmFeO_3_ (PDF#74-1474) with a high crystallinity. Three other diffraction peaks are present at 2θ = 28.254°, 32.741°, and 55.739°, which can be assigned to (222, 400) and (622), respectively; these results are consistent with the standard XRD patterns of Sm_2_O_3_ (PDF#42-1461). There is no peak for ZnO observed in the XRD spectra because of the low concentration of ZnO; however, in Fig. 2b, elemental Zn is clearly observed in addition to the elements of Sm, Fe, and O, which are also shown in the EDS mapping. No other diffraction peaks corresponding to impurities were observed, which indicated that the sample was a mixture of Sm_2_O_3_ and SmFeO_3_ with high purity.

**Fig. 3:**
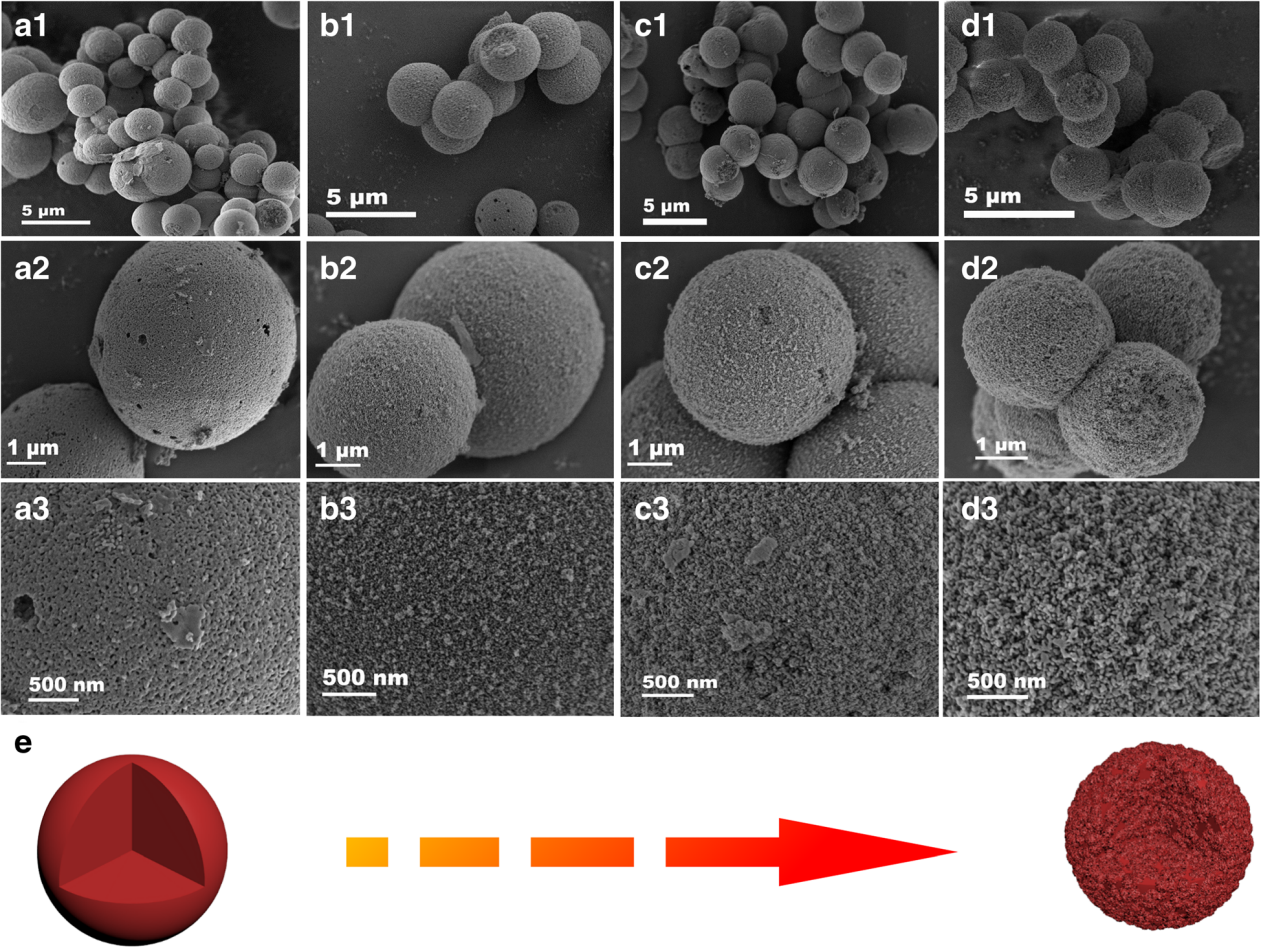
Different resolution SEM images of S1 (**a1**–**a3**), S2 (**b1**–**b3**), S3 (**c1**–**c3**), and S4 (**d1**–**d3**). **e** Schematic diagram of the formation process from S1 to S4

Low-magnification SEM images are shown in Figs. 3(a1–d1), which exhibit a panoramic of the as-obtained S1, S2, S3, and S4, respectively. As shown in the four images, the diameters of the obtained Sm_2_O_3_/ZnO/SmFeO_3_ microspheres were approximately 2–3 μm, and no other morphological characteristics indicated perfect uniformity or dispersibility of the samples. Figure 3(b1–b4) shows enlarged SEM images of the samples. As the hydrothermal time increased, the contact surface of the microsphere increased, which lead to the reduction of special sites on the surface.

Additionally, enlarged SEM images of the surfaces of S1, S2, S3, and S4 are shown in Fig. 3(c1–c4). The surfaces of the four samples were rough with a large number of nanoparticles. The space between neighboring nanoparticles was clearly visible especially in Fig. 3(c3) and Fig. 3(d3). This phenomenon indicates that the roughness increased as the time of hydrothermal treatment increased, which could result in an increase in specific surface area (Fig. 3e). The rough surface with pores considerably enhanced the specific surface area, which effectively improved the response due to the increased number of active sites. Combined with the conclusion of Fig. 3b and Fig. 3d, BET was necessary to define which sample had the largest surface area.

**Fig. 4:**
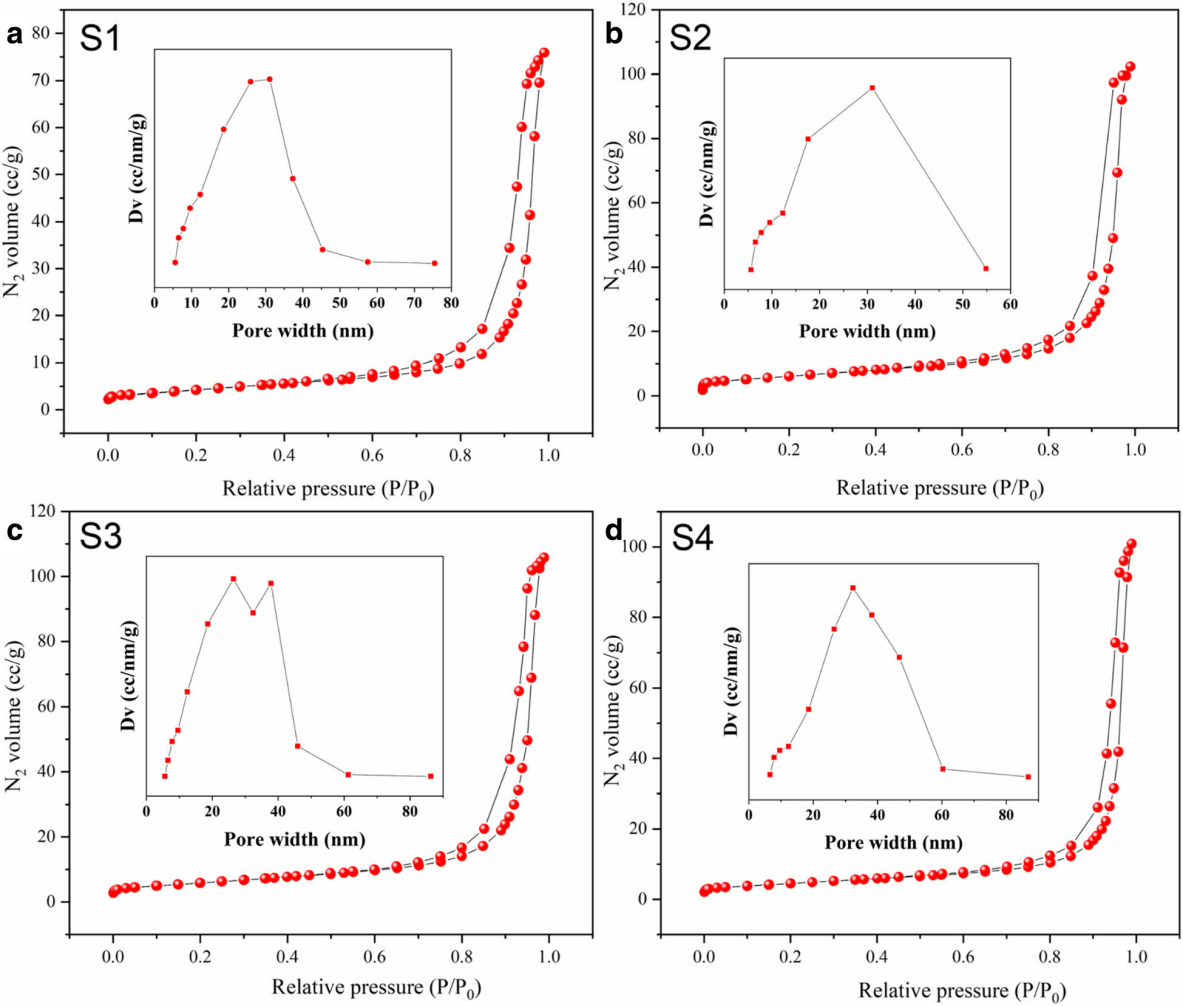
Nitrogen adsorption-desorption isotherms and corresponding pore size distribution curves of S1 (**a**), S2 (**b**), S3 (**c**), and S4 (**d**)

The specific surface area and pore volume are important factors for gas-sensing performance. Thus, the N_2_ adsorption-desorption isotherms were also measured, as shown in Fig. 4. As observed, the N_2_ adsorption-desorption isotherms of the four samples were indexed to the P/P_0_ axis, which represents a typical type-III isotherm with an H3 hysteresis loop [23]. N_2_ adsorption increased sharply when the relative pressure was *P*/*P*_0_ = 0.8. The two isotherms were almost linear at low pressure (0.2–0.8), which indicates that all samples had macroporous adsorption. The typical reversible isotherms indicate that all the samples exhibit slit-shaped pores. According to the pore size distributions, the average pore size was calculated to be 31.077 nm for S1, 31.046 nm for S2, 26.398 nm for S3, and 32.339 nm for S4 (Table 1.). The surface area was considerably influenced by hydrothermal time; the surface area of S3 was 27.579 m^2^/g, which was obviously higher than that of other samples (surface areas of S1, S2, and S4 were 21.159 m^2^/g, 26.150 m^2^/g, and 20.714 m^2^/g, respectively). The BET results are consistent with the sensing properties. A large surface area can provide more active sites and a large pore volume, enhancing the gas diffusion. As a result, the gas performance significantly improved.

**Table 1:** Textural properties of porous microsphere measurements of S1, S2, S3, and S4

Sample	Surface area (m^2^/g)	Average pore size (nm)	Pore volume (cm^3^/g)
S1	21.159	31.077	0.118
S2	26.150	31.046	0.156
S3	27.597	26.398	0.166
S4	20.714	32.339	0.156

**Fig. 5:**
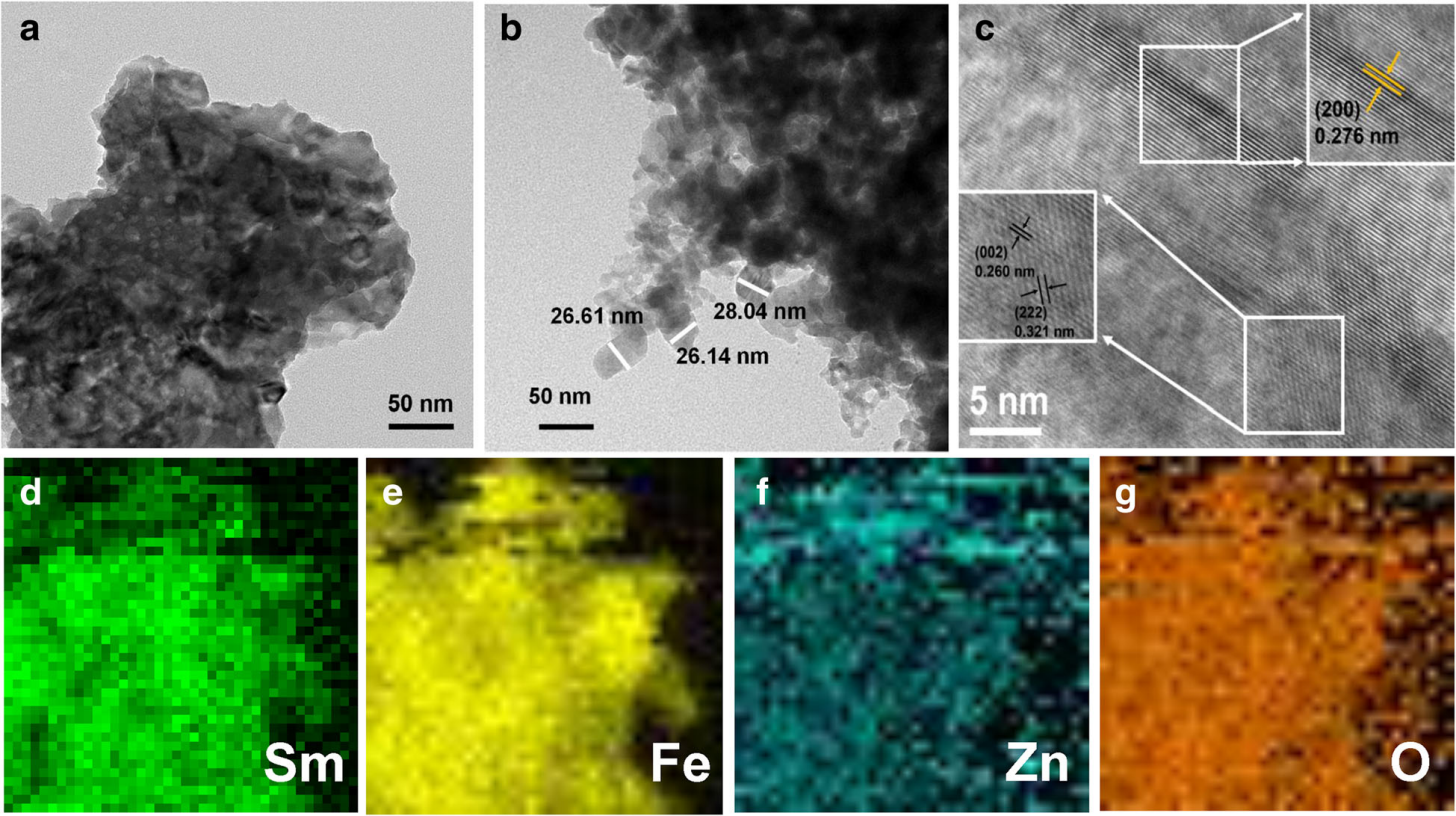
**a**, **b** TEM images and **c** HRTEM images of S3. STEM element mapping of S3 for Sm (**d**), Fe (**e**), Zn (**f**), and O (**g**)

S3 was chosen to further characterize because it had the largest surface area. The TEM image shows the structure of S3, which consists of nanoparticles with sizes of approximately 26 nm (Fig. 5b); this indicates that the microspheres were self-assembled by nanoparticles. The HRTEM investigation provided further insight into the structural features of the S3 microsphere, which is shown in Fig. 5c. The interplanar spacings were estimated to be 0.276 nm, 0.260 nm, and 0.321 nm, corresponding to the (200) plane of SmFeO_3_, the (002) plane of Sm_2_O_3_, and the (222) plane of ZnO, respectively (Fig. 5c inset). The element mapping in Fig. 5d, e, f, and g display the uniform distribution of Sm, Fe, Zn, and O, respectively. Clearly, the amount of Zn was relatively less than that of other elements.

**Fig. 6:**
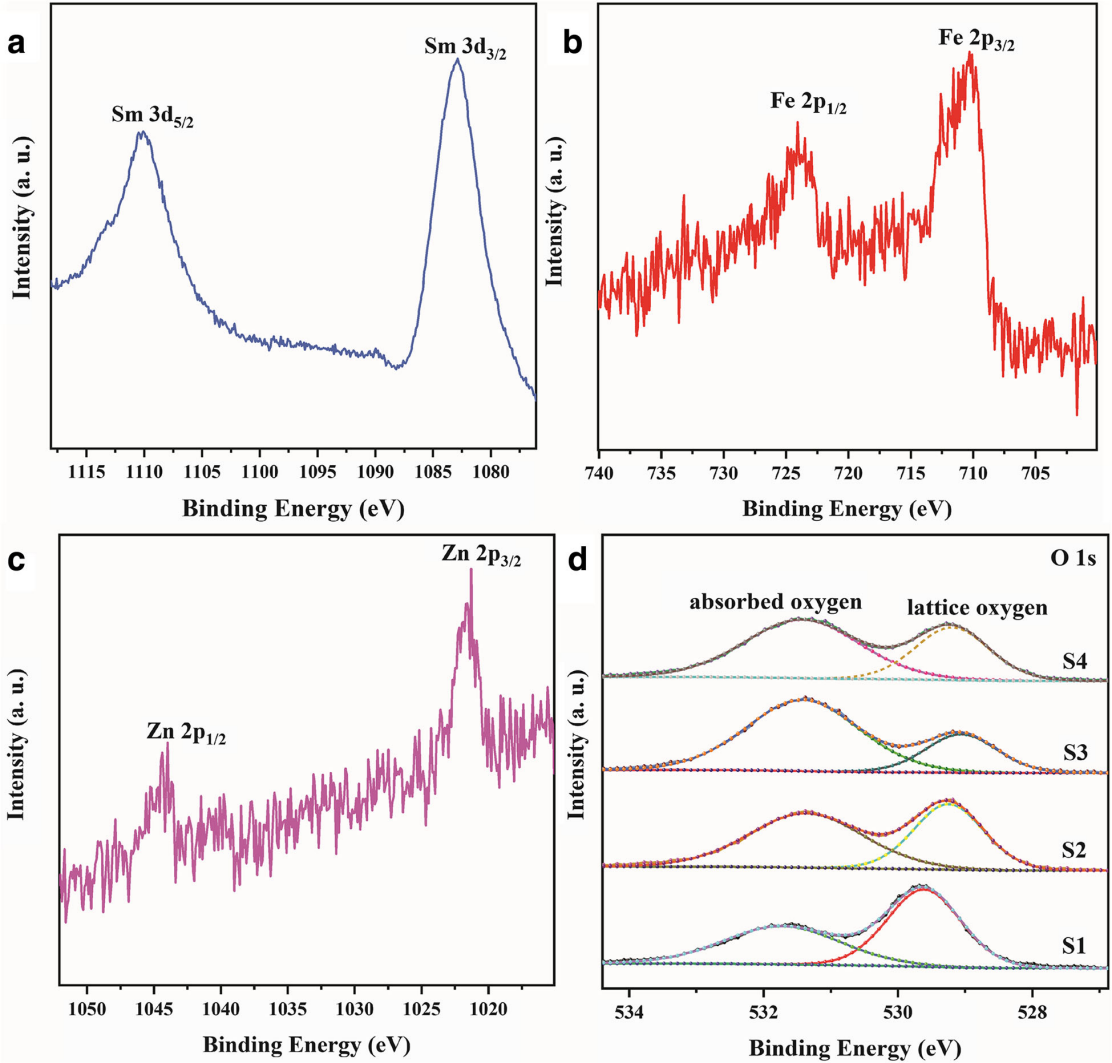
High-resolution XPS spectra of Sm (**a**), Fe (**b**), Zn of S3 (**c**), and O 1 s of S1, S2, S3, and S4 (**d**)

The XPS analysis of S3 is shown in Fig. 6. As shown in Fig. 6a, two peaks situated in 1082.9 eV and 1109.9 eV correspond to Sm^3+^ 3d_5/2_ and 3d_3/2_, respectively. Figure 6b displays the XPS spectrum of Fe 2p with peaks at 724.1 eV and 710.2 eV representing Fe^3+^ 2p_1/2_ and Fe^3+^ 2p_3/2_, respectively. The peaks at 1044.4 eV and 1021.3 eV are assigned to Zn^2+^ 2p_1/2_ and Zn^2+^ 2p_3/2_, respectively, confirming the existence of Zn^2+^ in the composite; this further confirmed the TEM results. The splitting of the 2p was 23.1 eV, which is in agreement with the energy splitting reported for ZnO and corresponds to the 2p binding energy of Zn (II). The absorbed oxygen species plays an important role in semiconductors in the gas-sensing process [24]. XPS analyses can confirm the ratio of adsorbed oxygen species; thus, high-resolution XPS of O 1 s for the samples was investigated, and the results are shown in Fig. 6d. As shown in Fig. 6d, there are two peaks attributed to O 1s. The peak at 531.4 eV corresponds to ${\mathrm{O}}_2^{-}$ in four samples, representing absorbed oxygen (${\mathrm{O}}_2^{-}$) on the surface of materials. Additionally, the chemical binding energies at 529.3 eV, 529.2 eV, 529.0 eV, and 529.2 eV correspond to lattice oxygen (O^2−^) in S1, S2, S3, and S4, respectively. Obviously, the O 1 s spectra reveal that the content of adsorbed oxygen of S3 is higher than that of S1, S2, and S4, which mainly attributed to the large surface area and different hydrothermal times. Different times for the hydrothermal reaction have huge effects on the amount of m-O (*m* = Sm, Fe, and Zn). A higher ratio of ${\mathrm{O}}_2^{-}$/${\mathrm{O}}^{2^{-}}$ can considerably enhance the gas-sensing performance [25]. In theory, a sensor based on S3 is a potential candidate material for a gas sensor.

## Results and Discussion

**Fig. 7:**
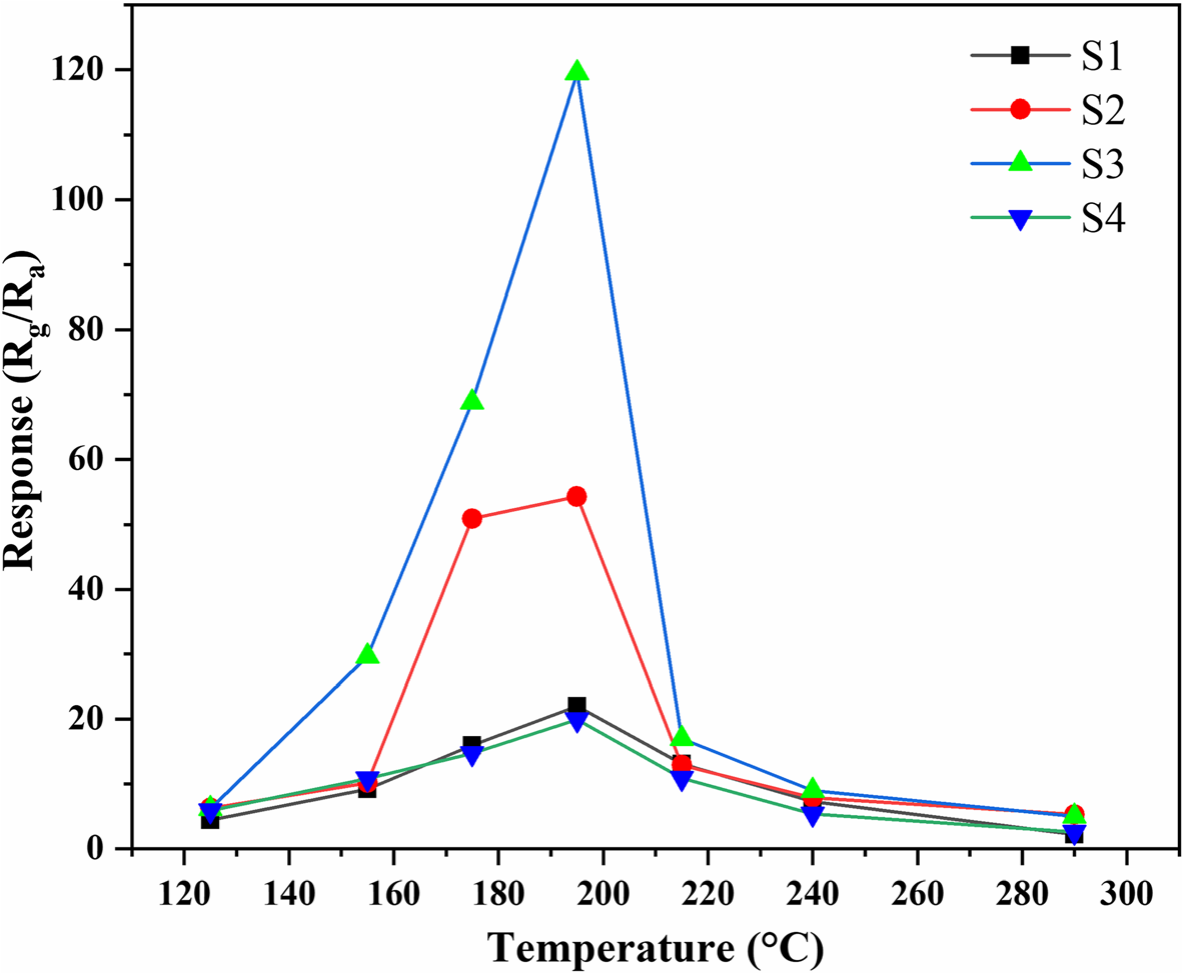
Relationship between response and operating temperature to 5 ppm methanol based on S1, S2, S3, and S4

Sm_2_O_3_/ZnO/SmFeO_3_ microspheres were synthesized as a potential sensing material for gas, and the gas-sensing performance of S1, S2, S3, and S4 were examined. In general, the responses of sensors are greatly influenced by temperature, and Fig. 7 shows the responses of S1, S2, S3, and S4 to 5 ppm of methanol measured at various operating temperatures (ranging from 125 to 295 °C). The maximum response values of S1, S2, S3, and S4 were 22.0, 54.3, 119.8, and 19.9, respectively, at 195 °C. The response of S3 was 5.4 times higher than that of S1, 2.2 times higher than that of S2, and 5.9 times higher than that of S4 at the same temperature. Therefore, 195 °C was chosen as the optimal operating temperature of the sensors for the following gas-sensing tests. At an operating temperature below 195 °C, the response significantly increased. In contrast, the response decreased as the operating temperature further increased. The responses of the sensors sharply increased with operating temperature at first, which was due to two reasons. First, the species of adsorbed oxygen changed with the operating temperature on the surface of the material. Second, as the temperature increased, the gas molecule could overcome the activation energy barrier of the surface reaction [26]. Afterwards, the response declined with increasing operating temperature. The reason for this phenomenon may be due to the drop in the number of methanol adsorption active sites with the increasing temperature. The other reason may be that the adsorption ability is lower than that of the desorption of methanol molecules, which leads to inferior performance of the sensing material at a high temperature. The S3 sensor exhibited a super high response to methanol gas, which indicates that Sm_2_O_3_/ZnO/SmFeO_3_ microspheres that undergo 24 h of hydrothermal time could be a potential methanol gas-sensing material.

**Fig. 8:**
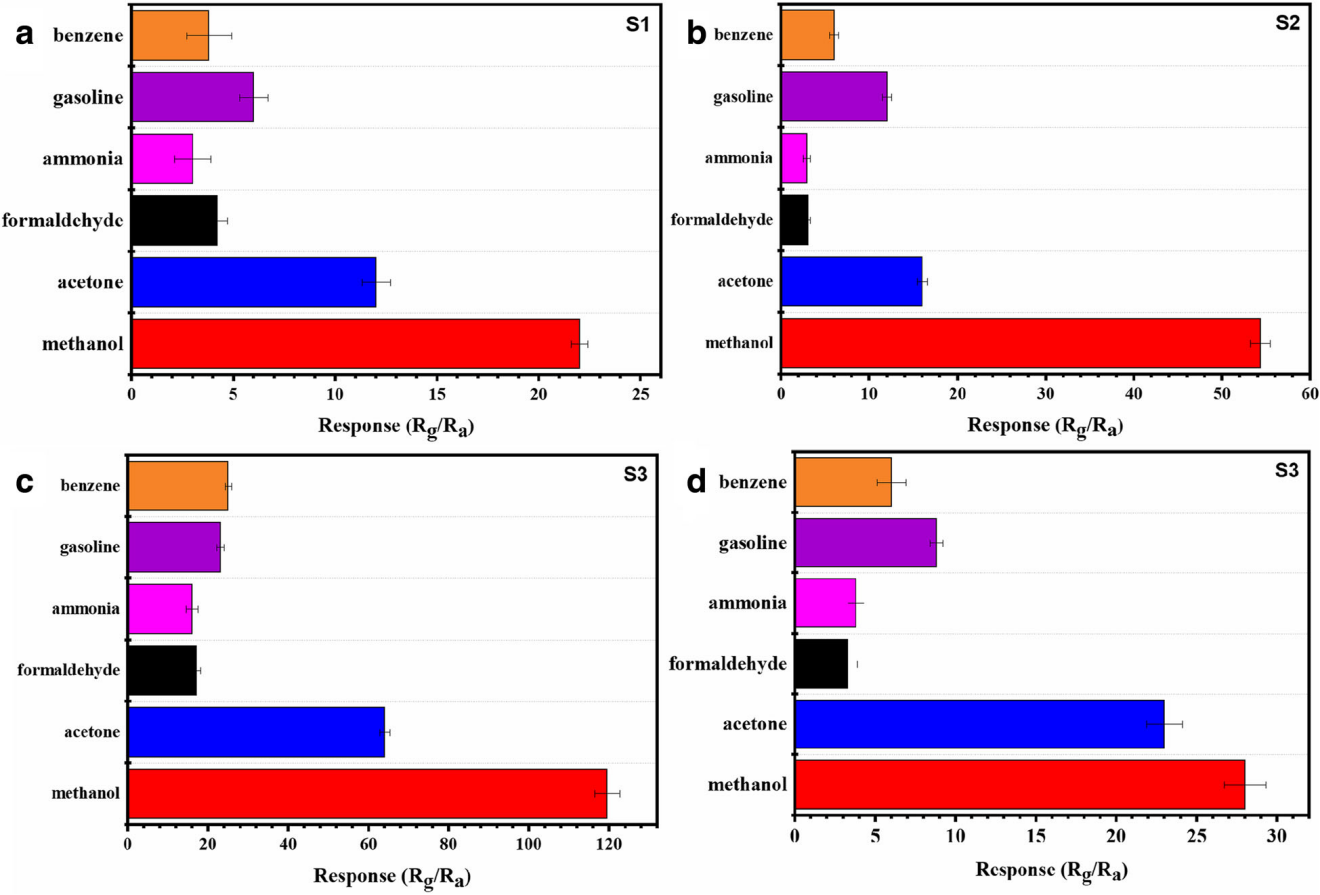
Selectivity of microsphere at different hydrothermal time based on S1 (**a**), S2 (**b**), S3 (**c**), and S4 (**d**) to various gases with a concentration of 5 ppm at 195 °C

To better distinguish methanol gas from other gases, the response to different gases at 5 ppm including acetone, formaldehyde, ammonia, gasoline, and benzene at 195 °C were measured to investigate the selectivity of S1, S2, S3, and S4 which are presented in Fig. 8a, b, c, and d. It can be observed that the response toward 5 ppm methanol is 119.8 while the response to acetone, formaldehyde, ammonia, gasoline, and benzene are 64.1, 17.2, 15.9, 23.0, and 24.8, respectively. The response gap between methanol and acetone reaches up to 55.7, it is high enough to discriminate other gases for a methanol gas sensor.

**Fig. 9:**
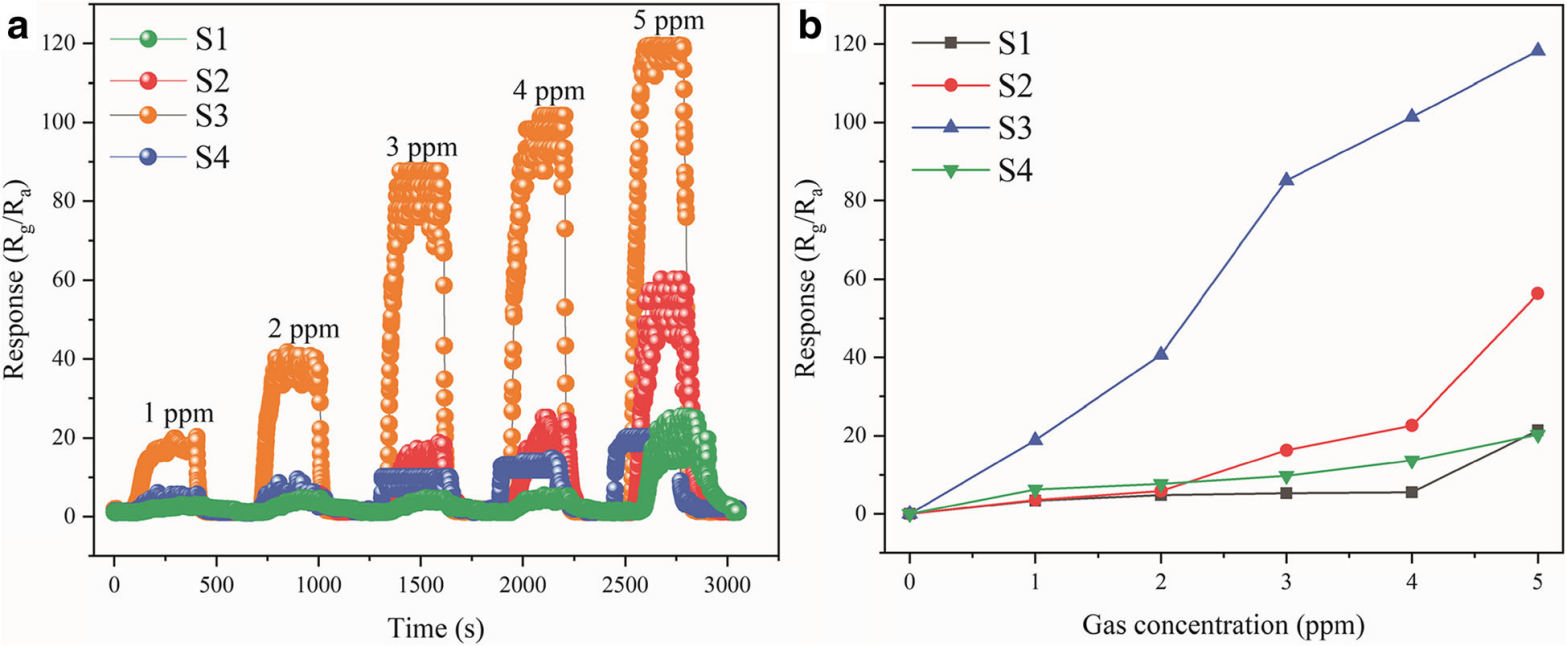
**a** Dynamical response transients of S1, S2, S3, and S4 to methanol gas at low concentration at 195 °C. **b** Relationship between response and concentration of S1, S2, S3, and S4 to different methanol gas concentration (1 ppm, 2 ppm, 3 ppm, 4 ppm, and 5 ppm) at 195 °C

Dynamical response transients of S1, S2, S3, and S4 to different methanol gas concentrations are displayed in Fig. 9a. As shown, the responses of S3 were approximately 19.8, 40.6, 85.2, 101.3, and 119.8 for methanol gas at 1, 2, 3, 4, and 5 ppm, respectively. Additionally, the other three sensors also showed response and recovery characteristics to different concentrations of methanol gas ranging from 1 to 5 ppm. There is a relationship between the response and concentration of the four sensors to methanol gas, as shown in Fig. 9b. The response of all sensors increased with increasing methanol gas concentration from 1 to 5 ppm; in particular, the response of S3 increased sharply with an increase in concentration. Obviously, the response significantly enhanced for S3 even at low concentrations of methanol (the response was 19.8 even at 1 ppm of methanol). The theoretical limit of detection is calculated via the least squares method [34]. According to the result of fitting in the linear regime, the slope is 25.24 and a fitting quality *R*^2^ = 0.972. One hundred thirty data were re-plotted points at the baseline of the sensor in the air; thus, using the root-mean-square deviation (RMSD) (1), the sensor noise can be calculated.

3 $$ {\mathrm{RMS}}_{\mathrm{noise}}=\sqrt{\frac{S^2}{N}}=0.0219 $$

The sensor noise is 0.0219 from the equation. The points were averaged and a standard deviation (S) was gathered as 0.062.

The theoretical limit of detection is approximately 7.37 ppb from Eq. (4):

4 $$ \mathrm{DL}=3\frac{{\mathrm{RMS}}_{\mathrm{noise}}}{S\mathrm{lope}}=7.37\ \mathrm{ppb} $$

**Fig. 10:**
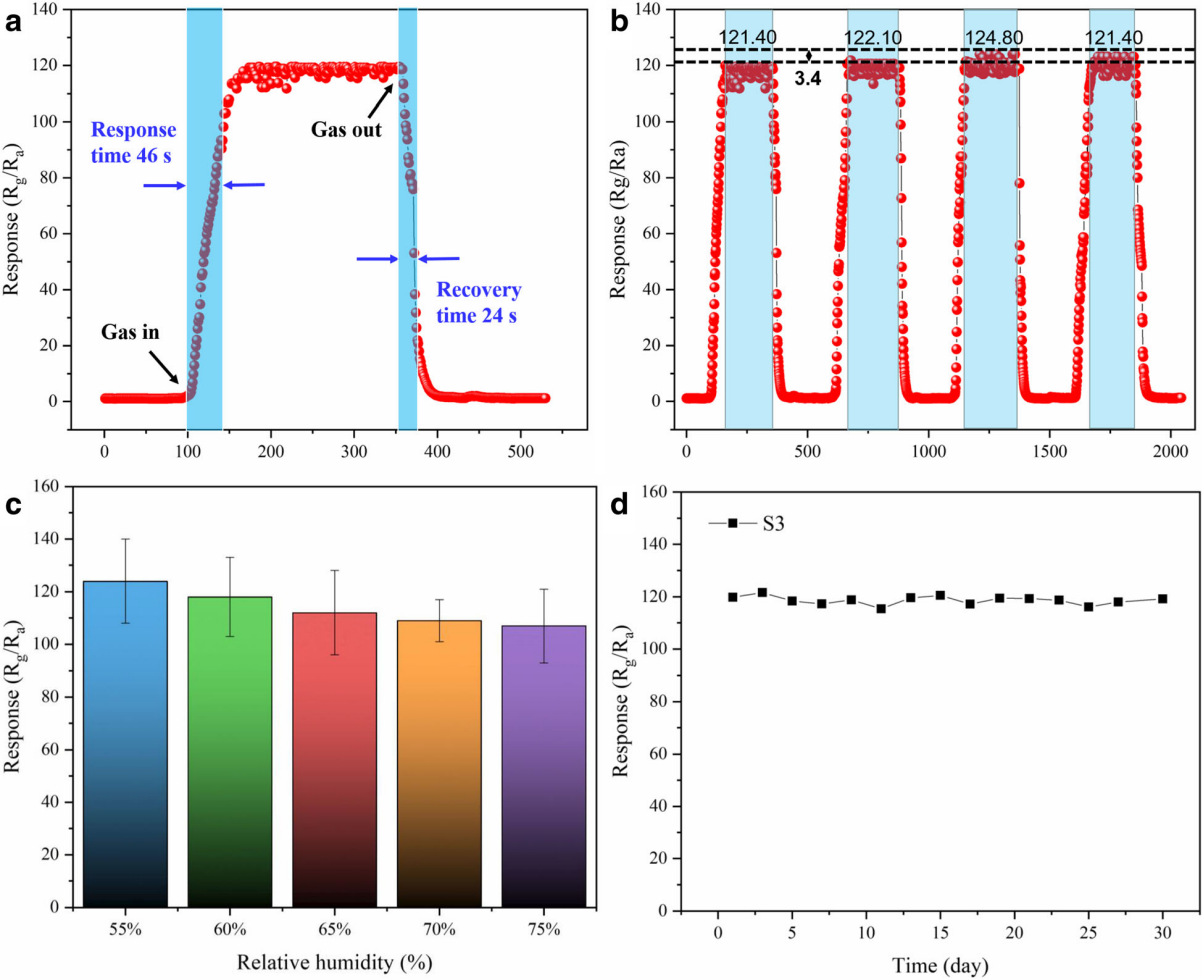
**a** Response and recovery curve of the S3 to 5 ppm methanol gas at 195 °C. **b** Reversibility of S3 to 5 ppm methanol gas at 195 °C under 4 cycles. **c** The relationship between response and relative humidity to 5 ppm methanol gas of S3. **d** Stability of S3 toward 5 ppm methanol gas for 30 days at 195 °C

The large surface area of S3 provides enough active sites to lead to a fast response. When the sensor was exposed to air, the response immediately descended to the original state. The time taken was only 24 s for this process, which was because of the desorption of methanol gas molecules and oxygen absorbed on the surface of the material. The reversible cycles and response (for 4 cycles) of S3 to 5 ppm of methanol gas at 195 °C was investigated, which is shown in Fig. 10b. The responses of S3 were 121.40, 122.10, 124.80, and 121.40 under the same conditions, which demonstrates the superior reproducibility of S3. To study the influence of humidity, the S3 response toward 5 ppm of methanol gas at 195 °C at a high humidity level was investigated, as shown in Fig. 10c. The responses of S3 to 5 ppm of methanol gas in 55% (RH), 60% (RH), 65% (RH), and 70% (RH) were 124, 118, 112, 109, and 107, respectively. The deviation in the response was only 17 in the range from 55 to 70% RH. The S3 gas sensor exhibited good stability even under a highly humid atmosphere, which indicated humidity-independent gas sensing for S3. The long-term stability of S3 to 5 ppm of methanol gas at 195 °C was measured (Fig. 10d). The response of the S3 sensor to 5 ppm of methanol at 195 °C in the 30-day test could be ignored. The excellent stability in the long-term was additional evidence for its application in industry.

Humidity interference is an important parameter for gas-sensing performance because the adsorption of water molecules may lead to less chemisorption of oxygen species on the surface [31]. Sm_2_O_3_ nanoparticles play a vital role in scavenging hydroxyl groups (OH) on the surface, maintaining a discernible response by assisting oxygen ion readsorption [35].

It is well known that the capacities of adsorbed oxygen species are closely associated with the gas-sensing properties of semiconducting oxides (Table 2). When the gas sensor works in ambient air, oxygen molecules absorb on the surface (${\mathrm{O}}_2^{-}$, O^−^, and ${\mathrm{O}}^{2^{-}}$) of materials and capture electrons, decreasing the electron concentration and increasing the hole accumulation layer of the surface material; this causes a drop in sensor resistance. As a typical p-type semiconductor exposed to an oxidizing gas, such as O_2_, the different types of oxygen species are different at different temperatures. The relationship between temperature and oxygen species is as follows [36]:

5 $$ {\mathrm{O}}_{2\left(\mathrm{g}\right)}\leftrightarrow {\mathrm{O}}_{2\left(\mathrm{ads}\right)} $$

6 $$ {\mathrm{O}}_{2\left(\mathrm{ads}\right)}+{e}^{-}\to {\mathrm{O}}_{2\left(\mathrm{ads}\right)}^{-}\left(<100{{}^{\circ}\mathrm{C}}\right) $$

7 $$ {O}_{2\left(\mathrm{ads}\right)}^{-}+{e}^{-}\to 2{O}_{\left(\mathrm{ads}\right)}^{-}\left(100{{}^{\circ}\mathrm{C}}-300{{}^{\circ}\mathrm{C}}\right) $$

8 $$ {O}_{\left(\mathrm{ads}\right)}^{-}+{e}^{-}\to {O}_{\left(\mathrm{ads}\right)}^{2-}\left(>300{{}^{\circ}\mathrm{C}}\right) $$

**Table 2:** Comparison of gas-sensing properties for methanol gas of various metal oxides with different morphologies

Sensing materials	Conc. (ppm)	*R*_methanol_	*T*_sens_ (°C)	Ref.
SnO_2_/ZnO	50	23	350	[27]
In/W ellipsoidal nanospheres	400	12	312	[28]
Ag-doped ZnO thin films	500	1.44	275	[29]
Co_3_O_4_	100	12	220	[30]
CeO_2_-decorated SnO_2_ hollow spheres	100	23.4	225	[31]
In_2_O_3_/CuO bilayer porous thin film	1000	2.9	250	[32]
Pd-WO_3_	10	32	350	[33]
Sm_2_O_3_/ZnO/SmFeO_3_ microsphere	5	120	195	This work

While the sensor is exposed to a reducing gas (such as methanol gas), the methanol gas molecules react with the absorbed oxygen on the material surface, and this will lead to electrons being released back to the semiconductor from adsorbed oxygen species, resulting in a decrease in the conductivity. The reaction between methanol gas molecules and adsorbed oxygen can be described as (9):

9 $$ {\mathrm{CH}}_3{\mathrm{O}\mathrm{H}}_{\left(\mathrm{gas}\right)}+3{\mathrm{O}}_{\left(\mathrm{ads}\right)}^{n-}\to {\mathrm{CO}}_2+{\mathrm{H}}_2\mathrm{O}+3{ne}^{-} $$

**Fig. 11:**
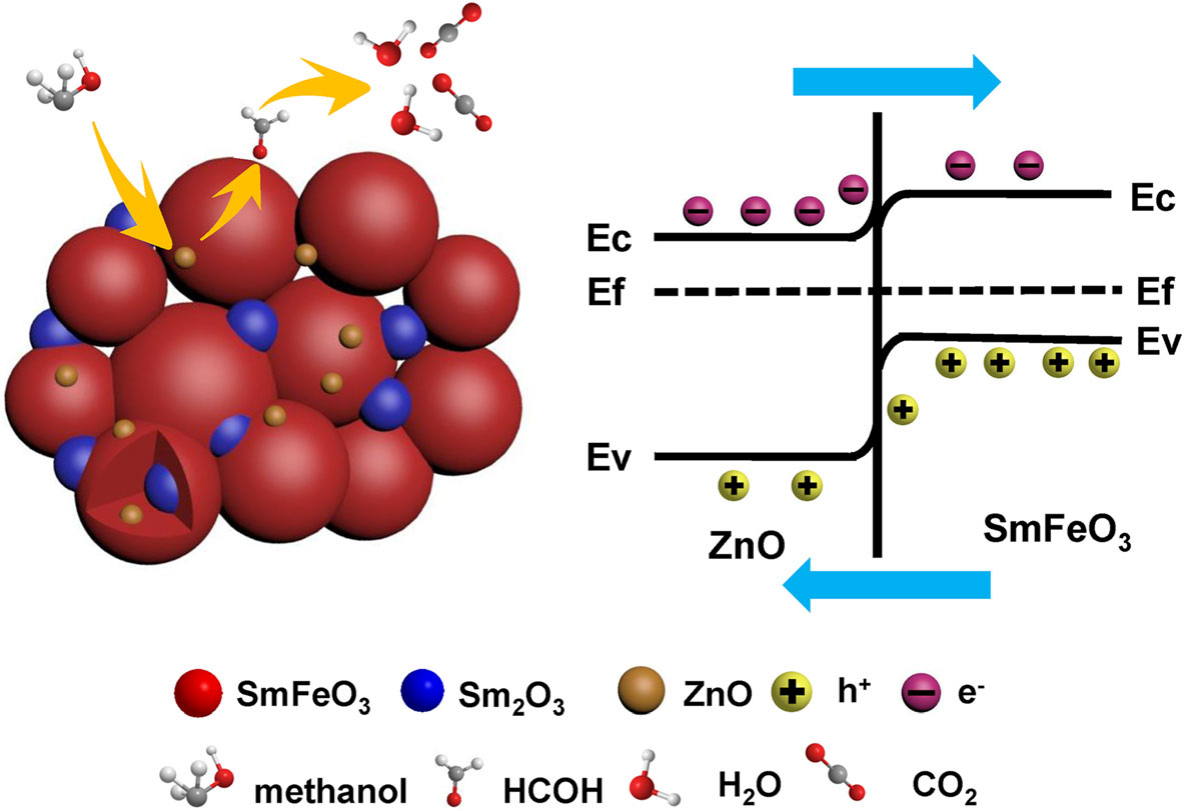
The schematic diagram of Sm_2_O_3_/ZnO/SmFeO_3_ p-n heterojunction

According to the above results, the S3 sensor showed excellent gas-sensing performance for 5 ppm of methanol gas. A schematic diagram of the Sm_2_O_3_/ZnO/SmFeO_3_ p-n heterojunction is shown in Fig. 11. The formation of a p-n heterojunction is one reason for the improved sensing properties. ZnO is an n-type semiconductor, and SmFeO_3_ is a p-type semiconductor, and upon combining ZnO and SmFeO_3_, a p-n heterojunction is formed between the surface of the two types of metal oxides. The electrons transfer from ZnO to SmFeO_3_, whereas the holes transfer to the opposite direction because of the different Fermi levels until a balance in the Fermi level and electron depletion layer emerges at the interface of the heterojunction [37].

The target gas (methanol) reacts with the adsorbed oxygen on the surface of ZnO, causing electrons to return back. The reactions at the interface of the heterojunction are shown in (10-11) [38]:

10 $$ {\mathrm{CH}}_3\mathrm{OH}+{\mathrm{O}}^{-}\left({\mathrm{O}}^{2^{-}}/{\mathrm{O}}_2^{-}\right)\to \mathrm{HCHO}+{\mathrm{H}}_2\mathrm{O}+{e}^{-} $$

11 $$ \mathrm{HCHO}+{\mathrm{O}}^{-}\left({\mathrm{O}}^{2-}/{\mathrm{O}}_2^{-}\right)\to {\mathrm{CO}}_2+{\mathrm{H}}_2\mathrm{O}+{e}^{-} $$

Additionally, the methanol gas with the hole in SmFeO_3_ produces the intermediate HCHO and furthers react with adsorbed oxygen on the surface of p-type SmFeO_3_ at the interface between the heterojunction (11–12):

11 $$ {\mathrm{CH}}_3\mathrm{OH}+{h}^{+}+{\mathrm{O}}^{-}\left({\mathrm{O}}^{2-}/{\mathrm{O}}_2^{-}\right)\to \mathrm{HCHO}+{\mathrm{H}}_2\mathrm{O} $$

12 $$ \mathrm{HCHO}+{h}^{+}+{\mathrm{O}}^{-}\left({\mathrm{O}}^{2-}/{\mathrm{O}}_2^{-}\right)\to {\mathrm{CO}}_2+{\mathrm{H}}_2\mathrm{O}+{e}^{-} $$

Therefore, the p-n heterojunction interface between the two types of metal oxides easily attracts reductive and oxidative gases. A deeper electron depletion layer will be formed, leading to an enhanced sensing performance.

In addition to the formation of a p-n heterojunction, the large specific surface and the high amount of adsorbed oxygen also attributed to improving the sensing performance. The order of specific surface area was S3 > S2 > S1 > S4, and the sensing responses of the four sensors were in the same order. This indicates that a large specific surface area is beneficial for sensing response, which provides more active sites for both the target gas and oxygen molecules and favors the surface catalytic reaction. S3 exhibits a higher ratio of ${\mathrm{O}}_2^{-}$/O^2−^ than S1, S2, and S4, and the results indicated that S3 had the highest ability for adsorbing ionized oxygen species, which may contribute to increasing the sensing performance [39].

## Conclusion

In this report, Sm_2_O_3_/ZnO/SmFeO_3_ microspheres were successfully synthesized as a methanol gas sensor, and we investigated the effect of different hydrothermal reaction times on the microstructure. The BET and XPS results reveal that different hydrothermal reaction times significantly influence the specific surface area and adsorbed oxygen species, which have a huge effect on the gas-sensing performance. The p-n heterojunction is another important reason for the enhanced performance. When the hydrothermal reaction time was 24 h, the sensor exhibited the highest performance for methanol gas. The response of the Sm_2_O_3_/ZnO/SmFeO_3_ microsphere reached 119.8 for 5 ppm of methanol gas at 195 °C in a relatively high humidity atmosphere, and the response was higher than 20 even at 1 ppm of methanol gas. In addition, the sensor also shows excellent repeatability and long-term stability only with a small deviation in the 30-day test. Therefore, a sensor based on Sm_2_O_3_/ZnO/SmFeO_3_ microspheres is a good choice for the detection of methanol gas.

## Abbreviations

BET: Brunauer-Emmett-Teller
DMFC: Direct methanol fuel cells
EDS: Energy dispersive X-ray spectroscopy
FESEM: Field-emission scanning electron microscopy
HRTEM: High-resolution transmission electron microscopy
MOS: Metal oxide semiconductors
PEG: Polyethylene glycol
RH: Relative humidity
TEM: Transmission electron microscopy
XPS: X-ray photoelectron spectroscopy
XRD: X-ray diffraction
